# Fighting for Masculine Hegemony: Contestation between Alt-Right and White Nationalist Masculinities on Stormfront

**DOI:** 10.1177/1097184X221120664

**Published:** 2022-08-16

**Authors:** Jillian Sunderland

**Affiliations:** 1 Sociology Department, 7938 University of Toronto, Toronto, ON, Canada

**Keywords:** white nationalism, masculinity, alt-right, anti-feminism, contestation, social movements, online, movement masculinity

## Abstract

The alt-right community serves as a gateway into the white nationalist movement. However, more research is needed on how the alt-right’s virulent misogyny interfaces with white nationalist masculinity premised on patriarchal protection of white femininity. This study addresses this question through a qualitative analysis of a white nationalist forum, Stormfront.org, and finds two masculine strategies vying for site dominance. These two gender strategies draw on different movement ideologies, white nationalist or alt-right. Users battle over the prime adversary used to construct movement identity and mobilize against. I argue forum conflict reveals that defining a central adversary is necessary for a masculine social movement to achieve a collective “movement masculinity” through a unification of goals and strategies. These findings contribute to research on masculinity and social movements by showcasing that not only is there diversity in extreme-right masculinity but that there is significant contestation over different masculine strategies.

## Introduction

The white nationalist movement has long been woven into the cultural fabric of the US, but the 2016 election brought a new political voice into the cultural lexicon: the “alt-right.” The alt-right has harnessed the internet to recruit adherents through eye-catching memes, humor, and trolling (Winter 2019; Grant and MacDonald 2020; Bezio 2018; Dignam and Rohlinger 2019). Through their multi-sited online presence, the alt-right has brought together formerly distinct cybergroups of white nationalists (DeCook 2018), the manosphere (Zuckerberg 2018; Van Valkenburgh 2021), and strings of the male gaming community known for their misogyny (Blodgett and Salter 2018). Prominent figures like Richard Spencer, a former Duke PhD student, labelled the “dapper” white nationalist, spoke in an eloquent manner urging whites to reclaim their country: “America was, until this last generation, a white country designed for ourselves and our posterity. It is our creation, it is our inheritance, and it belongs to us.” (Lombroso and Appelbaum 2016). He and others like Milo Yiannopoulos, a flamboyant self-described “gay jew,” are a far cry from the cloaked hoods and secrecy of traditional white supremacy.

Yet, even before the emergence of the alt-right, white nationalists were undergoing a transformation (Hawley 2018; Belew and Gutierrez 2021). In recent years, movement adherents toned down their rhetoric and strategically rebranded their message from “white supremacy” to “white nationalism” to gain a foothold in the Republican establishment. However, this political strategy has been a point of contestation for members of the alt-right as they largely reject this “politically correct” makeover and alignment with traditional conservatism (Hawley 2018). In a recent twitter post, Spencer told his 70,000 followers that “the GOP is now the “own the libs by being a moronic as conceivably possible” party” (Spencer 2021). Instead, the alt-right champions a strategy of shock and disruption through online harassment campaigns against women and minorities. Members of both camps tried to bridge this divide through the 2017 “Unite the Right” rally but failed in their attempt (Hemmer 2021).

Yet, the differing political strategies of the alt-right and the white nationalist movements mirror differences in their gender ideology. As such, I ask whether white nationalist masculinity has changed given the rise of the alt-right. To answer this question, I conducted a qualitative analysis of Stormfront.org, a leading white nationalist (WN) forum. Through my research, I uncovered two distinct gender strategies vying for site dominance. Forum users adopting a protective patriarchal strategy enact what I term *Aryan masculinity* formed in opposition to racialized men. Conversely, users who view women as their main “enemy” and consider themselves simultaneously dominant and victimized perform what I call *alt-misogynist masculinity.* The conflict between these two racist masculine strategies centers on the focal “other” against which movement identity is constructed—people of color or women. Bringing together scholarship on masculinity and social movement collective identity, I argue that agreeing on a central adversary is paramount to achieving what I denote as a collective “movement masculinity” that forms through working toward consensus on movement goals. These findings reveal, not only diversity in extreme-right masculinity, but significant conflict between gender strategies. I argue this illustrates how contestation can become central in developing masculine hegemony in social movement spaces.

## Literature Review

Scholarship on masculinities and social movements is critical to understanding white nationalist masculinity. Studies find many men’s movements are mobilized as a counter-response to feminism or widescale changes in the gender order (Messner 2016; Ging 2019; Leek and Gerke 2020; Dignam and Rohlinger 2019). Yet research uncovers that even movements focused on race, class, or sexuality are sites of gender construction. For instance, both Ferber (1998) and Blee (2008) argue white nationalist males organize around white masculinity. However, less attention has been paid to how a collective masculine identity is developed and sustained. Here, I first summarize existing scholarship on masculinities and social movement collective identity to show how both address the role of relational definitions and contestations in constructing group identity, which helps explain the formation of white nationalist masculinity. I further outline how this identity has been impacted by the rise of the alt-right and online anti-feminism.

Masculinity is not an “innate” identity but instead constituted through opposition to what it is not—principally femininity—but also through race, class, sexuality and more, creating hierarchies of masculinity (Connell 2005; Pascoe 2005). Hegemonic masculinity represents the apex of this power structure as the culturally-exalted strategy that legitimates gender inequality. Connell (2016) describes masculine hegemony as never settled but instead formed through symbolic battles as it is “struggled for, and struggled against, by different social forces” (306). Demetriou (2001) notes that when this conflictual process results in a unification of masculine strategies, hegemony can be achieved through collective opposition to a specified target. These masculine projects can be taken up at the local, regional, or global level as gender regimes are place- and context-specific (Connell and Messerschmidt 2005). One area in which these masculine projects take place is in online communities where men engage in collective identity work (Ging 2019).

Similar mechanisms are at work in social movement communities. Studies show members construct collective identity and a sense of “we-ness” by relationally defining themselves against other groups (Polleta and Jasper 2001; Rohlinger and Bunnage 2015; Beyer 2014). For instance, Carian’s (2022) study of a men’s rights activist (MRA) forum uncovers how posters mobilize a collective action frame in opposition to women and feminism, resulting in the creation of a misogynistic collective identity. Yet, like collective masculinity projects, creating a unified movement identity can be a conflictual process as “movements often fight internally over who is the focal antagonist” (Gamson 1997). Thus, both collective masculinities and collective movement identities delineate a focal “other” in identity construction work and engage in contestation.

### White Nationalism, the Family, and Relational Constructions of Masculinity

Research has found that white nationalist men form their masculinity in opposition to racialized men who are deemed “threats” to the family *and* white women who require protection (Bjork‐James 2020; Daniels 2016; Perry 2004). This construction is rooted in beliefs concerning the white nuclear family which both shores up their defender identities and underscores their racist positions. Seeing the family as the bedrock of white civilization, members believe that “the highest duty and honour of the white man… is to preserve the white family and with it a hierarchy of race, gender, and sexuality” (Daniels 2016, 39). Further, as they consider the gendered division of labor representative of *proper* social order, adherents view protecting the family as defending patriarchal social arrangements (Bjork-James 2020).

Through the symbolism of the family, white nationalists conceptualize white women as innocent and vulnerable, underpinning their protective patriarchal identity (Belew 2018; Bjork‐James 2020; Daniels 2016; Ferber 1998; Latif et al. 2020; MacLean 1994; Perry 2004). Blee (2002) notes in the post-antebellum era, the KKK used notions of imperiled white womanhood as a rallying cry for recruitment as “The symbols of white female vulnerability and white masculine potency took power equally from beliefs in masculine [power] and in white supremacy” (16). While modern white nationalists have toned down their rhetoric to appeal to more mainstream audiences, Hughey (2011) argues protecting white women is still used to define themselves as valorous protectors.

White nationalist men construct men of color as “threats” due to the latter’s purported hypersexuality, criminality, and degeneracy, while relationally defining themselves as strong, capable, and heroic (Bjork‐James 2020; Daniels 2016; MacLean 1994; Perry 2004). These constructions became crystallized in the Reconstruction-era myth of the “Black Male Rapist” that spurred the castrating and lynching of thousands of Black men to emasculate and eliminate them. These atrocities were not just based on protecting women but also aimed at preserving white male’s proprietary ownership of women and maintaining the “proper relations” between white and racialized masculinities. As Perry (2003) states, “The vicious forms of punishment meted out to Black males served to highlight their animal nature at the same time as reinforcing the power and hegemony of white males” (86). Modern white nationalists still use “threats” from racial others as a discursive mechanism to construct valorized masculinities.

### White Nationalism and the Alt-Right in Digital Space

While white nationalists previously did identity work through print publications and real-world social interactions, since the 1990’s the internet has become their main tool in developing a collective “we-ness” (Winter 2019; Daniels 2016; Bjork James 2020; DeCook 2018; Bliuc et al. 2019). During the early stages of online activism, WN leaders simply disseminated propaganda, reflecting a top-down approach that maintained distinct cybercommunities (e.g., white supremacists, neo-Nazis, militia). But with the advent of interactive websites, members were able to participate and interconnect more freely, making the sites more democratic and the boundaries between groups more porous (Daniels 2009; Bjork-James 2020). However, some studies find that the fluid nature of “open structure” online movements can have a negative effect on the development of collective identity as they can lead to infighting over the movement’s mission and focal enemy (Buyukozturk, Gaulden, and Dowd-Arrow 2018; Rohlinger and Bunnage 2015; Beyer 2014; Daniels 2009). Yet, the emerging alt-right movement capitalized on this new interconnectivity as it offered them a larger audience to espouse its platform.

Since 2010, the alt-right has leveraged the internet to draw people into white nationalism, quickly becoming trendy on online sites like Reddit, 4chan, 8chan, Twitter, and YouTube (Hawley 2018). It successfully exploited the interconnection on the web and brought together several distinct cybercommunities—white nationalists, gamers, men’s rights activists, pickup artists, incels—under one banner. Participation in one subgroup often led members to interact with and embrace more extreme communities. The manosphere, a collection of anti-feminist websites, has had particular success in recruiting converts to WN by encouraging men to take the “red pill,” and awaken to the “truth” about society (Jones et al. 2020; Dignam and Rohlinger 2019; Ging 2019; Schmitz and Kazyak 2016; Carian 2022). Analyzing 300 million comments on Reddit and YouTube, Mamié, Ribeiro, and West (2021) not only find overlap in these communities but that users who start out consuming MRA content frequently migrate to white nationalist spaces. This connectivity between both groups has also affected the manosphere. Both Ging (2019) and Zuckerberg (2018) note many sites in the manosphere now incorporate explicitly white nationalist content showing the porous boundaries between the alt-right and white nationalist movements. Due to a crossover of membership, I explore whether this new digital landscape has made constructing a cohesive WN movement masculinity more elusive.

## Data and Methods

Data were collected from Stormfront.org, the longest-running online white nationalist Web site created by KKK leader Don Black in 1995 (Törnberg and Törnberg 2021, 288). Initially, Black posted essays by leading white nationalists including William Pierce, author of *The Turner Diaries*—a book that inspired the Oklahoma City bombing. The lasting endurance of Stormfront has been due to his transforming the site into a digital community and forum in 2001 (Daniels 2009; Hartzell 2020). Presently, the community boasts a membership of 330,000, including notable white supremacists Edward Field, Thom Robb, and David Duke and has over 30,000 daily guest visits, primarily from the United States (Bjork-James 2020; Bliuc et al. 2019; Winter 2019; Hartzell 2020). The average user age increased from 31 in 2001 to 42 in 2016, reflecting the site’s success in retaining long-term users committed to the cause (Törnberg and Törnberg 2021). This large and far-reaching number of subscribers and guests has proved deadly as Stormfront has been connected to over 100 murders (Beirich 2014).

I chose Stormfront as it is specifically for “white nationalists” (WNs) and its message-board style gave me access to decades of posts where users debate topics and share experiences. Not only does it “offer a behind-the-scenes glimpse into the construction of white nationalism” (Hatzell 2020, 132), it reveals the “contested inside life” of the movement (Törnberg and Törnberg 2021; Hartzell 2020) that is typically unavailable through static hate sites where administrators post public-facing comments (e.g., American Renaissance, The Daily Stormer). The forum allows rank-and-file WN members to create discussion threads to which other users may post comments. Many threads remain active for years with new posts challenging past claims and reigniting interest. As such, these threads serve as an ongoing archive of movement beliefs and goals and any contestation around them.

I treated forum comments as my documentary data in line with qualitative document analysis (QDA). QDA is particularly generative for this project because it facilitates the “immersion, exploration, and contextual understanding” of mediated textual data (Altheide et al. 2008, 134). Given the challenges associated with ethnography, “ethnographic” document analysis^1^ of forum posts offers an alternative means of accessing the internal workings of the group. In light of the sheer volume of threaded discussions, I set specific parameters on my data collection (Gerstenfeld, Grant, Chiang 2003) and sampled threads from the Ideology and Philosophy section, determining it most salient to the movement’s ideological framework and identity formation. Following QDA protocol, I performed purposeful sampling guided by my research questions as this is the recommended strategy for managing web-based data (Gerstenfeld et al. 2003; Hester and Dougall 2007; Scheibling 2020). I selected threads based on predetermined keywords appearing in titles (e.g., “masculinity,” “alt-right,” “family”). This strategy generated 4867 comments in 87 threads from April 2010 – April 2021 collected over a 2-year period (March 2019 – April 2021). During this time of sustained engagement with the site, I analyzed all new posts under existing threads and new threads to reduce bias. I saved the threads as PDFs which I imported into NVivo, a qualitative analysis software.

I undertook an inductive analysis starting with careful reading of user discussions and preparing reflective memos to aid in identifying salient topics (Altheide et al. 2008; Thomas 2006). I then created descriptive codes that I used in the first round of coding (Saldaña 2021). I began to see recurring thematic topics in the posts that were then consolidated into a set of three primary codes: (1) the family; (2) control; (3) Movement goals. Based on ideological differences I identified within each main code, I created sub-codes. For example, differences in how the family was perceived such as “family is the bedrock of civilization” as opposed to “family benefits women” gave rise to the secondary codes: “sanctity of family” and “family problematic.” In total, I developed eight secondary codes associated with “family,” seven secondary codes associated with “control,” and six secondary codes associated with “movement goals” (see table 1).

**Table 1.** Breakdown of Main Codes.*

	Aryan Masculinity	Freq	%	Alt-Misogynist	Freq	%
		2462	59		1717	41
						
Family		986			740	
						
	Sanctity of family	378		Family problematic	196	
	Family vs. POC+	337		Family versus women	280	
	Nature/Together	75		Nature/Cruel	179	
	Jewish threat	196		Feminism threat	85	
						
Control		576			433	
						
	Self-control	247		Control others	203	
	Vs. racial other	152		Vs. women	90	
	Jew’s control us	177		Women control us	112	
				Racial flexibility	28	
						
Movement goals		900			544	
						
	Include women	179		Include Alt	157	
	POC as enemy	552		Women as enemy	263	
	Aryan against Alt	169		Alt against white night	124	
						
Total					4179	

* In line with some CDA approaches, I created frequencies of occurrences to better contextualize my data. Codes specifically related to racism are not reflected in the table as they were not deemed salient for my analysis of masculine strategy.

+ “POC” includes Jewish People as WN see them as non-white.

Notably, there was much contestation on the forum around the three primary themes: family, control, and movement goals. Through analysis of these conflicts, I identified the presence of two distinct groups espousing differing ideologies—white nationalist and alt-right. These groups also appeared to be divided along generational lines (typically older “boomers” and younger “millennials”). The contestation seemed to revolve around the issue of the movement’s main “enemy” or “other.” Informed by scholarship on masculinity, I began conceptualizing and coding the groups as two discursive configurations of masculinity. I created sub-themes to tease out the different viewpoints. For instance, I coded “We’re not like Blacks who can’t form families” as family versus POC under the theme of “family” and under the category of Aryan masculinity. The proportion of these “otherings” is reported in table 2**.**

**Table 2.** Proportion of Oppositional Definitions.

Aryan Masculinity	Vs. POC	1041/62%
Alt-Misogynist Masculinity	Vs. women	633/38%

## Findings

The dominant gender strategy, which I term “Aryan masculinity,” projects a protective patriarchal identity reflecting traditional white-nationalist beliefs around women and family and is reflected in 59% of posts. In contrast, users adopting alt-misogynist masculinity enact an aggressive and domineering masculine strategy rooted in alt-right ideologies, particularly the manosphere’s misogyny. This strategy appears in 41% of posts that attacked women, expressed men’s victimization, depicted marriage as a mere social contract, and promoted enforced gender roles and women’s (violent) subjugation. I argue that what lies at the heart of these masculine gender strategies is competing definitions of the “other” that reveal differing ideologies around race, class and, most importantly, gender. Those enacting Aryan masculinity construct their identity mainly in opposition to racialized men, as reflected in 62% of coded posts, while alt-misogynists use women in 38% of coded posts (table 2).

While social movements need to construct a “we-ness” and agree on goals to advance their political agenda, the divergent focus of these users leads to battles over which masculine strategy should be adopted, mirroring Connell’s notion that gender hegemony is always subject to contestation. Conflicts coalesce around three main themes: marriage, family, and gender relations; control; and movement goals.

### Marriage, Family, and Gender Relations

Family is a central topic of discussion on Stormfront as revealed in several threads such as “Being a Homemaker and Housewife is a respectable profession” and “Would you like to be part of a movement that helps white families?” A cursory view seems to confirm Simi and Futrell’s (2006) finding that Stormfront users have a strong propensity to “idealize conservative traditional patriarchal family forms and community relations dominated by Aryan kinship” (117). My analysis, however, uncovered family ideologies as more complex and diverse. Both Aryans and alt-misogynists are united in seeing the white nuclear family in peril and both draw on biological essentialism to endorse and preserve this traditional family form. However, they diverge on who or what is a threat to the family and the essentialist arguments that underpin them. Aryans are adamant that Jewish people want to destroy the white family, prompting “racial warrior” rhetoric (196 posts). In contrast, alt-misogynists view feminism as a threat and see males as under attack by a “gynocentric” society (85 posts). These beliefs are shored up by essentialist arguments about the family. Aryans believe traditional gender roles and marriage are “natural” and immutable and, as such, the traditional family form will prevail (75 posts). Alternatively, alt-misogynists see natural law as conspiring against them and argue women’s hypergamous “nature” disadvantages beta males like them (179 posts). The higher proportion of alt-misogynists’ “nature is cruel” posts reveal how extensively misogynistic discourse has taken hold in this online space. These viewpoints become crystalized in each groups’ discursive gender strategies as both use a differing “other” to bolster their masculinity. While Aryans claim they embody the hegemonic norm of heading up families by contrasting themselves against racialized men who they contend fail to form families (337 posts), alt-misogynists view women as preventing men from assuming this hegemonic role, prompting them to adopt compensatory behaviors (280 posts).

For Aryans, the family serves as a symbolic marker for race and gender supremacy as reflected in the post, “Families are the building blocks of White civilization.” According to Bjork James (2020), WNs draw on Enlightenment Era thought and thus view the nuclear family as essential for nation building and proper social order through its instillation of hierarchical and ordered unions. WNs’ veneration of the family is reflected in the “14 words” epithet which expresses their goal of securing the future of the white race by creating a new white homeland populated by white families (Bjork James 2020). Thus, any threats to the family represent a threat to white civilization itself. This belief is often reiterated as the racist conspiracy of “white genocide” which claims Jewish people are bent on eliminating the white race by promoting inter-racial sexuality and homosexuality. As one user states, “(((The Powers That Be))) are doing everything they can to pit men and women against each other, but nature wants us to be together.” Here, the supposed threat of Jewish people is represented via triple brackets, an antisemitic symbol adopted by WNs. However, for this Aryan user, this threat is mitigated by a belief in the essentialist argument that “nature” wants white men and women to be together.

Yet for Aryans, the family does not only crystalize their racist ideologies but also serves as a mechanism to construct their masculinity. As relational definitions lie at the heart of masculinity (Connell 2005), Aryans define themselves as successful husbands and fathers in contrast to men of color who they contend fail to live up to these hegemonic norms. One user asks, “Did you ever see a black family with a father? Please make a photo, because it’s very rare. Not being a father is a sign of a highly immature being.” As Townsend (2010) conceptualizes, modern manhood refers to a “package deal” which includes marriage, employment, homeownership, and fatherhood. For Aryans, the “package” of patriarchal manhood is also a racial norm only white men can fully embody. Disparaging Black men as absent fathers, unemployed, etc. allows this poster and fellow Aryans to shore up this hegemonic norm for themselves.

Alt-misogynists have a different orientation to marriage and fatherhood. Rather than considering these ways to achieve normative masculinity and racial superiority, they are disillusioned and commonly reference being shut out of the “package deal” due to feminism. Their gender essentialist ideologies reinforce this belief leading them to air grievances that women are innately programmed to mate with strong, good-looking, and successful “alpha” males and reject men like them (Zuckerberg 2018). One user posted, “Women will instantly betray their own tribe, the moment their hardwired instincts tell them the invading tribes are stronger and more suitable for childbearing. ….that’s Nature and you can’t change it.” The idea that women are naturally prone to hypergamy and “trading up” is pervasive in the manosphere and reflects the use of evolutionary psychology to support misogynistic rhetoric. Ging (2019) found this viewpoint prompts men to react in divergent ways: striving to achieve alpha status (pickup artists), disengaging from the whole process (men going their own way), and adopting violent orientations (incels- involuntary celibates).

Because alt-misogynists view women as predisposed to reject or discard them, women become the prime adversary in constructing their masculinities. As such, they attempt to reclaim their patriarchal dividend either through subordinating wives/partners or avoiding marriage and accruing masculine capital in other ways. According to one user wary of women’s intentions, “That’s why you force them into a two-hundred-page signed prenup with an expensive hired attorney… any kind of relationship I get into I’m going to be the one having all the advantages or dominance not the other way around.” As Zuckerberg (2018) and Van Valkenburgh (2021) note, this transactional view of intimate relations, where dominance is something to be fought and won, forms the basis of MRA mindsets. When domination is not possible, users seem to disengage from the prospect of marriage while affirming their supremacy. One user claimed, “Women are more than willing to throw their husbands away for the slightest grievance…The only men that get married now are desperate or foolish.” Here this user “recuperates” from expected rejection by inverting the popular notion that single men are lonely, immature, and lack a sense of responsibility. These users thus frame married men as the desperate and foolish ones. As such, alt-misogynists engage in “compensatory manhood acts” (Schrock and Schwalbe 2009) as maligning women allows them to believe they are not “lesser” men who can’t get women, but are, in fact, superior.

Yet, alt-misogynists’ anti-woman attitude is contested on the forum as Aryans see this as incompatible with the WN goal of countering “white genocide” through the formation of white families. In a thread titled “How women weaken nations (and why men let them)” two users engage in a discursive power battle over which masculine strategy should be dominant, mirroring Connell’s (2005) assertion that masculinities are prone to contestation as men strive to achieve hegemony.

[alt-misogynist:] *You older men don’t know or even grasp how terrible things have become for the 35 and younger crowd*. I don’t hate women, I hate what they’ve become. *Many women have become the enemy* as they’ve become weaponized against the west and men alike….

[Aryan:] There are no hordes of men who hate women the way you describe. It’s more like a few guys who can’t get laid.... *So they join that mgtow and whine* to high heaven about how bad women are. So for the third time, for the most part, men and women get along just fine… The vast majority of men and women will continue to date, get married, have families and live happy lives together. *It’s instinctive, it’s nature, it’s normal.*

*As for being an old guy*...I admit I don’t like some of the changes….But at the end of the day women are still our partners in life.

[alt-misogynist:] In other words *you’re still stuck in the 1960’s* where you’re not at all interested in learning about other people’s opinions or insights.

This discussion focuses on the principal “enemy” of the movement with the alt-misogynist attempting to convince the Aryan that women are responsible for men’s current plight. According to Dignam and Rohlinger (2019), men in the manosphere adopt a strategy of denigrating and blaming women to bond with other men. However, this strategy fails here as the alt-misogynist is disparaged by the Aryan who invalidates the other’s masculinity by claiming their anger arises from an inability to “get laid.” Notably, these users attribute their differing positions to a generational schism that has resulted in different outlooks on marriage and family. Messner (2016) argues these generational divides can be attributed to “historical gender formations” that result in differing orientations to the gender order.

### Control

Since users believe in the racist conspiracy that whites are being eradicated through immigration and inter-racial sexuality, controlling women’s sexuality and curtailing “race-mixing” becomes paramount. Moreover, control informs cultural constructions of masculinity more generally and is thus a prevalent theme (Schrock and Schwalbe 2009, 280). While there is consensus that men now have less control over their lives and women, Aryans and alt-misogynists disagree on the target of blame and strategies to re-assert control. Aryans blame racial others, particularly Jewish men and the Zionist-organized government (ZOG), for their loss of control (177 posts). Contrastingly, alt-misogynists blame women and feminism for their lack of control over their lives (112 posts). These different orientations foster disparate discursive strategies for regaining control. While Aryans champion a self-control strategy that they believe sets them apart from racialized men (152 posts), alt-misogynists propose violently taking back control from women (90 posts).

Aryans affirm that to wrest back control they must first practice self-control, willpower, and self-development to earn the respect of others. One user posted, “As a masculine man you determine what you want in life and accomplish it using your willpower.” As Schrock and Schwalbe (2009) argue, a central feature of masculinity is exercising control over self and resisting control by others, which is paramount for this user. Yet, this Aryan strategy should not be taken at face value as it masks an underlying purpose—maintaining dominance over women. As stated by another user, “The mentality that is productive…is taking ownership of the responsibility for becoming the kind of man to whom a woman would *submit*.” MacLean (1994) argues that the KKK’s emphasis on “honor” and “chivalry” had the implicit purpose of controlling women’s chastity and securing obedience. As such, these discourses are part and parcel of signalling a masculinity strategy that facilitates the movement goal of controlling women’s sexuality to populate a white homeland.

However, for Aryans, this self-control strategy is also a way to proclaim racial superiority as they contrast themselves against men of colour. As one user asks, “Are blacks masculine? …Blacks are violent because they cannot control themselves...That’s masculine, right? White men should beat more women to prove they are masculine.” Here, racialized men’s violence is believed to be due to a lack of control and their need to engage in “compensatory manhood acts” to assert dominance (Ezzell 2012). Users opine that it is only white men who can enact hegemonic notions of control based on consent and legitimization, while racialized masculinity is associated with violent domination.

Opposingly, alt-misogynists argue dominance over women needs to be restored by any means, including physical violence. Blaming women for male emasculation, one user claims that “Women have men thoroughly locked down and spiritually controlled, confused, compliant, exploited.” Alt-misogynists frequently bemoan their lack of sexual success with women and attribute their lack of job and promotional opportunities to feminism. In recent years, young men have grappled with being shut out of traditional avenues for achieving hegemonic success. Employing misogynistic rhetoric has thus been a way of expressing these frustrations for many men (i.e., Grant and MacDonald 2020; Kelly 2017). For members of the alt-right, this has led to their endorsing and engaging in violent tactics. The most violent expression of this reactionary formation is seen in the incel community which has been linked to several misogynistic mass murders like the 2018 Toronto Van Attack (O’Donnell and Shor 2022). Preceding the attack, a Facebook post stated: “The Incel Rebellion has already begun!” referencing the online trope that “beta” men would revolt and stake revenge on women who reject them and control the sexual marketplace that disadvantages them (Bates 2021).

Rather than eschewing violence to distinguish themselves from men of colour, alt-misogynists are willing to align with racialized men to dominate women, even expressing views antithetical to WNs central precept—racial supremacy. As one user commented, “Our Muslim brothers have been showing us how to treat femoids.” This comment not only presents a stereotypical view of Muslim men as violent but reflects a common masculine strategy of forming intra-gender bonds to exert dominance over women (Demetriou 2001). Further, using the dehumanizing term “femenoid,” (Preston, Halpin, and Maguire 2021), this user signals allegiance with the incel community highlighting the alt-right’s ideological elasticity (Ging 2019). While promoting total subjugation of women (i.e., “white sharia”) only appeared in 28 posts, this notion is surprising to find on a WN forum.

However, alt-misogynists’ anti-women views and proposed strategy to wrest control from women received significant pushback from Aryans. The following exchange in a thread titled “*Acceptably Aryan Masculinity”* highlights the conflict over the best way to take back control, one premised on exercising self-control, the other on using aggressive tactics.

[Aryan:] I want to make it clear that a man harassing or needlessly haranguing a white woman is an abominable act that should be met by any Aryan man with said stoic indignation.

[alt-misogynist:] *But White women are so out of control* they need a lot of *haranguing*.…. We seriously need a paradigm shift here. .. I may not post any more in this thread, because, so long as you are in that paradigm, we won’t see eye to eye on anything, and this discussion won’t go anywhere.

[Aryan:] Too many young males have been taught to view chivalrous behavior as “weak” and the *pimp game* as the epitome of masculinity…. A man doesn’t need to be domineering to be dominant. Domineering behavior is a product of insecurity.

In this exchange, both the alt-misogynist and Aryan recognize their viewpoints cannot be reconciled. The alt-misogynist claims women are so “out of control,” that hardline intimidation is needed to re-establish men’s dominance. The Aryan disparages this “domineering” style of masculinity as an illegitimate form of masculine authority, mirroring Connell’s (2005, 77) contention that “it is the successful claim to authority, more than direct violence, that is the mark of hegemony.” As studies show, seeking validation of masculinity is important for many men (Ezzell 2012; Schrock and Schwalbe 2009) but here the alt-misogynist’s manhood is delegitimized. The Aryan’s use of the term “pimp game” is significant as it is a racialized marker of subordinate masculinity that they claim “young males” have adopted. Here users again highlight a generational divide whereby a younger generation of alt-misogynists rejects the paternalism of older white nationalists. Instead, the alt-misogynist argues a different “paradigm” is necessary to reinvigorate the movement, especially as it relates to women’s place in the movement.

### Movement Goals: The Way Forward

The future direction, goals, and how best to grow the movement is also a significant topic of contestation on Stormfront. Throughout movement history, this has been a recurring theme. In the 1950s, several pro-white groups (e.g., White’s Citizens Council) went to great lengths to differentiate themselves from the violent KKK (Feinberg 2017). Similarly in the 1980s, white nationalists distanced themselves from young neo-Nazis who doubled down on extreme violence (Belew 2018). Recently Feinberg (2017) finds that after the Unite the Right rally Stormfront users distanced themselves from the event claiming it was orchestrated by the alt-right.

Similar divides over strategy and movement goals are reflected in my analysis. I find that Aryans promote the view the movement should try to attract more women into the fold (179 posts) and unite in opposition to racial others (552 posts). This signals their explicit rejection of merging with the alt-right (169 posts). Contrastingly alt-misogynists claim the movement should join arms with the alt-right (157 posts) and lambast Aryans for being “white nights” (124 posts) who fail to recognize women as a main threat (263 posts). Thus, a collective vision for the movement is hindered by irreconcilable differences over who constitutes the ‘other’ or enemy of the movement and who can serve as allies in the fight to preserve the white race.

Aryans argue that encouraging women to join would increase their numbers and facilitate movement goals. One user opines, “I think the best thing we can do is create virtual communities which are more attractive for women….Another thing to consider is how feminists recruit women, maybe we could learn a few things from them.” Viewing women as allies reflects earlier white supremacist practices of having women play a more central role in movement activities. During the 1920s, the KKK created a women’s auxiliary and wives of Klansmen organized family outings (Belew 2018; Blee 2002; Latif et al. 2020). Incorporating women into ethno-nationalist community building is also seen in the white nationalist recruitment hashtag “#TradWives” that circulates on Tik Tok, Instagram, and YouTube. In videos and blog posts, young WN women encourage viewers to have white babies to address low birth rates in the West and counter the supposed “white genocide” (Kelly 2018). A user known as “Wife With a Purpose” gained international attention with her white baby challenge: “I’ve made six! “Match or beat me!”

Aryans’ aim of including women in the movement also feeds into their race-focused conception of masculinity. These users consider their (supposed) egalitarian views and paternalistic masculinity as antithetical to the misogyny and violence they associate with men of color: “The non-white men saw women as mere possessions and slaves throughout history, whereas we white men saw them as our mothers, sisters, kin and wives, and should remain so.” By projecting onto racialized men anachronistic gender views, they define themselves in opposition as racially superior, moral, and righteous, highlighting the centrality of relational definitions in constructions of masculinity and gender hegemony.

In contrast, alt-misogynists see women as impediments to movement goals and that forum users should instead reach out to the alt-right. One user stated, “It’s too late for many indoctrinated white women. And our race is going to take a substantial hit in the coming years because of this.” Rather than potential allies, women are seen as exacerbating the problem of the declining white majority in Western nations. Many posts by alt-misogynists specifically blame women for the progressive (liberal) politics of today as reported in Törnberg and Törnberg (2021) that found more anti-women sentiment on Stormfront after Obama’s victory. Opposed to the #TradWives recruitment tactic, many in the alt-right turn even on white women who are trying to help the cause calling them “Trad Thots.”

Although some in the alt-right have called for less misogyny,^2^ I argue virulent misogyny is common on Stormfront as women are the central foils against which they define their masculinity: “Creating civilization is masculine. Destruction of civilization is feminine. That is why you see women succumb to degeneracy much easier than men.” Instead of reaching out to women, alt-misogynists propose Stormfront members should form inter-movement bonds with men associated with the alt-right: “I would recommend this site do more to attract the alt-right. *It’s foolish to write off an entire racialist youth movement.*”

The different stances on movement allies and enemies becomes a hot topic of debate on Stormfront. In a thread titled, “The alt-right versus boomers’ debate in summary*,*” younger users express their opinion that white nationalism has become stagnant due to the mentality of “Boomers” and advocate for a “paradigm shift” that would align them with the alt-right. They also complain their views are not respected by site moderators and older members, again highlighting the generational divide:

“So, if I was to be unfortunately shown the door it would only be because the *older more conservative minded mods* here simply can't accept ideas outside of their republican thought bubbles. *The type of ideas that would fundamentally change the direction white nationalism has been headed in since the end of the 20th century.*”

This user accuses moderators of acquiescing to traditional Republican thought that many members in the broader alt-right movement denounce (Dignam and Rohlinger 2019; Hawley 2018). The proposed “change” the alt-misogynist is advocating for is the replacement of pastoral protective masculinity with a masculinity strategy based on forceful domination of women.

This battle over the focal enemy and collective vision comes to a head when, in a rare move, site creator Don Black attempts to shut down this contested discussion by affirming movement goals can only be achieved by white men and women working cooperatively.

“Keeping Stormfront alive thirty years has taken too big a toll on my family and me, and it’s now a greater liability than ever before, just so I can provide a platform for your pseudonymous drivel.... I’m surprised you haven’t espoused the *Incel/MGTOW sickness prevalent among much of the ‘AltRigh*t.’ ‘White women are worse than Jews,’ you know… *Stormfront has and always will remain focused on our real enemies. Winning this war will take White men and women of all age groups working together.*...But you and your tagteam trollmates have become nothing but toxic. And, unfortunately for you, I own the board.”

Through this post, Black sends an implicit warning to younger members that their anti-women sentiment is antithetical to the movement’s “real” enemies—Black, Jewish people and racial minorities—and may lead to their removal from the forum. His disparaging reference to “Incel/MGTOW sickness” also reveals he would prefer a smaller ideologically-coherent movement than a larger movement divided over misogynistic sentiment. These posts highlight the fracture within this social movement space and reveal the Aryans’ local dominance is maintained through the backing of moderators.

## Discussion

Two discursive strategies of masculinity vie for dominance on Stormfront. The first and most dominant masculinity, *Aryan masculinity*, embraces traditional tenets of white nationalism: racial superiority and patriarchal protection of white women and families. The second masculinity, *alt-misogynist masculinity,* is a regressive, domineering, and vitriolic enactment that draws heavily from alt-right ideology, especially the manosphere and incel communities. Users’ conflicts coalesce around posts relating to gender and family; masculine control; and movement goals. The underlying factor giving rise to these two configurations is conflict over a different focal “other” that relationally defines these masculine strategies.

The importance of a central adversary in these two strategies reveals that lying at the heart of masculinity constructions are relational definitions of what they are not. While all masculinities refract onto various people/groups (Hughey 2011; Connell 2016), I argue this takes on central importance in masculine social movements. As Demetriou (2001) notes, there must be a unification *within* masculinities for groups to impose external hegemony, which involves the suppression of other masculinities or an amalgamation of traits and strategies defined against a target. Coalescing around a central target is also necessary for the formation of a movement identity (Rohlinger and Bunnage 2015; Beyer 2014) to achieve consensus on movement goals. Based on my findings, I argue that a central task of masculine social movements is constructing a unified “movement masculinity” defined in opposition to a mutually agreed-upon focal enemy.

As discussed, however, Stormfront users are divided over the “other” which creates a political fracture within the movement, evidenced by the considerable contestation on the forum. While social movement studies of the feminist movement have examined the impact of internal conflict (Winch, Littler, and Keller 2016), I show how discord impacts masculinist movements, specifically impeding the construction of a unified WN masculine identity. My study thus builds on research that has documented diversity in extreme-right masculinities (Ging 2019; Schmitz and Kazyak 2016; Jones, Trott, and Wright 2020) by uncovering the effects of contestations and conflict within these spaces.

The conflict I found on Stormfront supports Connell’s (2016) assertion that masculinity construction and hierarchical structuring follow a cyclic historical pattern such that dominant masculinities are continually challenged and replaced by other styles. Through analysis of a “local” community (Connell and Messerchmidt 2005) where this plays out, I found that self-described younger alt-misogynists waged a discursive battle to wrest power away from the older Aryan users. However, Aryans maintained their dominance by capitalizing on their majority position, having the support of site moderators, citing their long-time commitment to white nationalism, and employing discursive strategies aimed at invalidating alt-misogynistic masculinity. While King et al. (2021) argue that older men seem to consent to their declining masculine status, I found these older users fought to maintain dominance over younger members.

My analysis also reveals the presence of two generational cohorts that come head-to-head over issues best reflected in the thread “Alt-Right versus Boomers debate in summary*.*” During conflicts, users frequently narrated how differences in the social-political climate in which they came of age has resulted in divergent ideologies. Younger members claim older users are totally “out of touch” with today’s realities – high divorce rates, fewer job prospects, and increased equality for women. Preston, Halpin, and Maguire (2021) and O’Donnell and Shor (2022) find young men reference these realities as leading to their adoption of incel ideology, which I also found on Stormfront. However, older Aryans did not appear to be as impacted by recent transformations in the gender order. Thus, I argue transformations within the gender structure may not prompt a refashioning of *all* masculinities but seem dependent on men’s relative historical positioning. Given the generational divide on the forum, future research should explore generational cohorts as an entry point to studies of masculinities.

My findings lead me to conclude that scholars studying masculinities should pay greater attention to the struggle for power between them. Seeing how masculine hegemony is contested and fought over, even in local settings, may reveal insights into the production of hegemony more broadly.

## Notes

1. Altheide et al. (2008) describes an ethnographic component to QDA.

2. See Marcus Follin and “The Woman Question” in Kelly 2018.
